# Incidence of intraoperative hypotension in older patients undergoing total intravenous anesthesia by remimazolam versus propofol: A randomized controlled trial

**DOI:** 10.1097/MD.0000000000036440

**Published:** 2023-12-08

**Authors:** Yeong-Gwan Jeon, Sujin Kim, Ji-Hyoung Park, Jonghoon Lee, Sang A Song, Hyun Kyo Lim, Seung Woo Song

**Affiliations:** a Department of Anesthesiology and Pain Medicine, Wonju College of Medicine, Yonsei University, Wonju, Republic of Korea; b Department of Anesthesiology and Pain Medicine, Wonju Severance Christian Hospital, Wonju, Republic of Korea.

**Keywords:** anesthesia, hypotension, intraoperative complications, intravenous anesthetics, propofol, remimazolam

## Abstract

**Background:**

An increase in the frequency of surgeries among older individuals is observed in some countries. Hypotension is common and exaggerated in older patients and can lead to increased morbidity and mortality. Total intravenous anesthesia is commonly administered with propofol, while remimazolam has been suggested as an alternative to propofol because of advantages such as a more stable hemodynamic profile and less respiratory suppression. We conducted a single-blind, parallel-group randomized controlled trial to compare the incidence of intraoperative hypotension between patients administered with remimazolam and propofol.

**Methods:**

A total of 132 patients, aged between 65 to 80 years and undergoing laparoscopic cholecystectomy or transurethral resection of bladder tumors were randomly assigned to the propofol or remimazolam group with a permuted block system while being blinded to the hypnotic agent. Remifentanil was administered via target-controlled infusion in both groups, with an initial effect-site concentration of 3.0 ng/mL and titration range of 1.5 to 4.0 ng/mL intraoperatively. The primary outcome of this study was the overall incidence of hypotension during general anesthesia.

**Results:**

Patients in the propofol group experienced higher intraoperative hypotension than those in the remimazolam group (59.7% vs 33.3%, *P* = .006). Multivariate logistic regression analysis showed that remimazolam administration was associated with reduced hypotension (adjusted odds ratio, 0.34; 95% CI, 0.16–0.73). Secondary outcomes such as recovery time, delirium, and postoperative nausea and vomiting were comparable in both groups.

**Conclusion:**

Total intravenous anesthesia with remimazolam was associated with less intraoperative hypotension than propofol in older patients, with a comparable recovery profile.

## 1. Introduction

In high-income countries, a surge in the incidence of the older population undergoing surgeries is observed.^[1,2]^ Increased left ventricular afterload caused by arterial stiffening and reduced baroreceptor function is a frequent complication in older patients.^[3]^ Exaggerated hypotension is common and should be avoided during surgeries in these patients.^[2]^

Total intravenous anesthesia provides better quality of recovery and enables intraoperative monitoring of the somatosensory or motor-evoked potentials.^[4–6]^ Propofol is the most commonly administered hypnotic agent for total intravenous anesthesia.^[4]^ Remimazolam, a novel short-acting benzodiazepine, is an alternative hypnotic drug to propofol. Hypotension is reportedly less common in patients administered with remimazolam than in those administered propofol.^[7]^ This study compared the incidence of hypotension in older patients undergoing total intravenous anesthesia with remimazolam versus propofol.

## 2. Methods

### 2.1. Study setting

This study was designed as a single-blind, parallel-group, randomized controlled trial and was conducted in a tertiary university hospital in the Republic of Korea from December 2021 to April 2023. This study was reviewed and approved by the Institutional Review Board of Wonju Severance Christian Hospital (CR321106, approval date: October 14, 2021). This study was registered with the Clinical Research Information Service, Republic of Korea (KCT0006719, registered on November 08, 2021) and reported in accordance with the Consolidated Standards of Reporting Trials guidelines. Candidates for enrollment were informed about the study via an informed consent form. Only those candidates that provided informed consent were considered and the procedures were conducted in accordance with the Helsinki Declaration.

### 2.2. Participants

Patients undergoing general anesthesia were enrolled if they fulfilled the following inclusion criteria: age between 65 to 80 years, elective surgical schedule of transurethral resection of bladder tumor (TURBT) or laparoscopic cholecystectomy, and American Society of Anesthesiologist physical status classification ≤ III. Exclusion criteria were as follows: ambulatory surgery, unable to measure noninvasive blood pressure on the brachial arteries and invasive blood pressure monitoring on the radial arteries, chronic alcoholics, body mass index ≥ 30, hepatic failure of Child-Turcotte-Pugh Class C, acute narrow-angle glaucoma, a history of hypersensitivity to any trial drugs, and inability to understand the informed consent form. The criteria for withdrawal were the expression to discontinue participation or a suspected hypersensitivity reaction.

### 2.3. Study protocol

After obtaining informed consent, one of the authors (JL) randomly assigned the participants to the remimazolam or propofol group using a permuted block system. Random allocation sequences were generated for TURBT and laparoscopic cholecystectomy by one of the authors (YGJ) using R statistics 4.2.2 (R Core Team, Vienna, Austria). The size of the permuted block was 6, and the permuted block was generated independently for each surgery to maximize comparability. The allocation of the last 6 participants was adjusted to equalize the number of enrolled patients in both groups.

The patients were blinded to group allocation. The hypnotic drug was prepared after the patient arrived in the operating room and the infusion device was placed outside the patient’s sight. Blinding was maintained for 24 hours after the conclusion of surgery.

No premedication was administered to any participant, and they underwent standard anesthesia monitoring with a bispectral index (BIS) monitor upon arrival at the operating room. In the remimazolam group, the rate of remimazolam administration was 6 mg*kg^-1^*hours^-1^ for loss of consciousness (LOC). After LOC, the remimazolam administration rate was adjusted between 1 − 2 mg*kg^-1^*hours^-1^ to maintain a BIS of 50. In the propofol group, infusion was performed via the target-controlled infusion (TCI), using the Schnider model. Propofol was administered at an effect-site concentration of 4.0 µg/mL.^[8,9]^ After LOC, propofol administration was regulated between 2.5 to 4.0 µg/mL to maintain a BIS of 50.

Remifentanil infusion was administered via TCI in both groups using the Minto model. Infusion was started with an effect-site concentration 3.0 ng/mL with the hypnotic agent and titrated in range of 1.5 to 4.0 ng/mL following endotracheal intubation. Rocuronium was administered 0.8 mg per kg ideal body weight for neuromuscular block. Additional rocuronium 0.15 mg per kg ideal body weight was administered when there was a request from the surgeon for visual field improvement, surgical manipulation, or > 20% of abrupt reduction of lung compliance despite appropriate respiratory management.

Ephedrine 8 mg was administered intravenously to treat the hypotension. Phenylephrine infusion was commenced where ephedrine could not resolve hypotension for 3 times at this dose in 1 event. Further treatment was determined by an attending anesthesiologist where hypotension persisted beyond this point.

Ramosetron 0.3 mg was administered upon initiation of skin suturing, and the infusion of remifentanil and hypnotic drugs was ceased 5 minutes prior to the conclusion of surgery. Sugammadex was administered as a neuromuscular reversal agent. Flumazenil 0.2 mg was administered to patients in remimazolam group whose recovery of spontaneous ventilation was inadequate 10 minutes after cessation of the remimazolam infusion.^[7]^

### 2.4. Variables and assessments

The primary outcome of this study was the overall incidence of hypotension during general anesthesia. Hypotension was defined as systolic blood pressure < 90 mm Hg.^[10,11]^ The number of hypotensive events were recorded.^[12]^ Secondary outcomes were postoperative nausea and vomiting, delirium, cardiac arrest, or death within 24 hours after surgery and bradycardia ( < 60 beats per minutes) or tachycardia ( > 100 beats per minutes) from the induction of anesthesia to postanesthetic care unit discharge. Delirium was assessed 24 hours after the conclusion of surgery in accordance with the DSM-V criteria.^[13]^

### 2.5. Statistical analysis

Per protocol analysis was performed for the primary outcome and other categorical variables using the chi-square test. As the number of intraoperative hypotensive events presented a non-normal distribution, they were compared using the Wilcoxon rank-sum test. Other continuous variables were compared using t-tests. Statistical significance was set at *P* < .05. Missing values were excluded from the analysis.

Binomial logistic regression analysis for intraoperative hypotension was performed to compare the risk of hypotension with that of hypnotic drugs and adjust for the effects of covariates. The covariates included in the analysis were surgical type and American Society of Anesthesiologist physical status classification.^[14]^ The R statistics 4.3.1 program was used for statistical analysis and visualization.

### 2.6. Sample size

A previous study compared the incidence of hypotension between patients administered with remimazolam and propofol as anesthetic drugs, and hypotensive events occurred in 60% of the patients administered propofol and 34.7% of those who administered remimazolam.^[15]^ Setting statistical power and statistical significance at 0.80 and 0.05, with assumption of a similar incidence of hypotension, 60 participants were required in each group. Considering a 10% withdrawal rate, 132 participants were enrolled in this study.

## 3. Results

**Figure 1.**
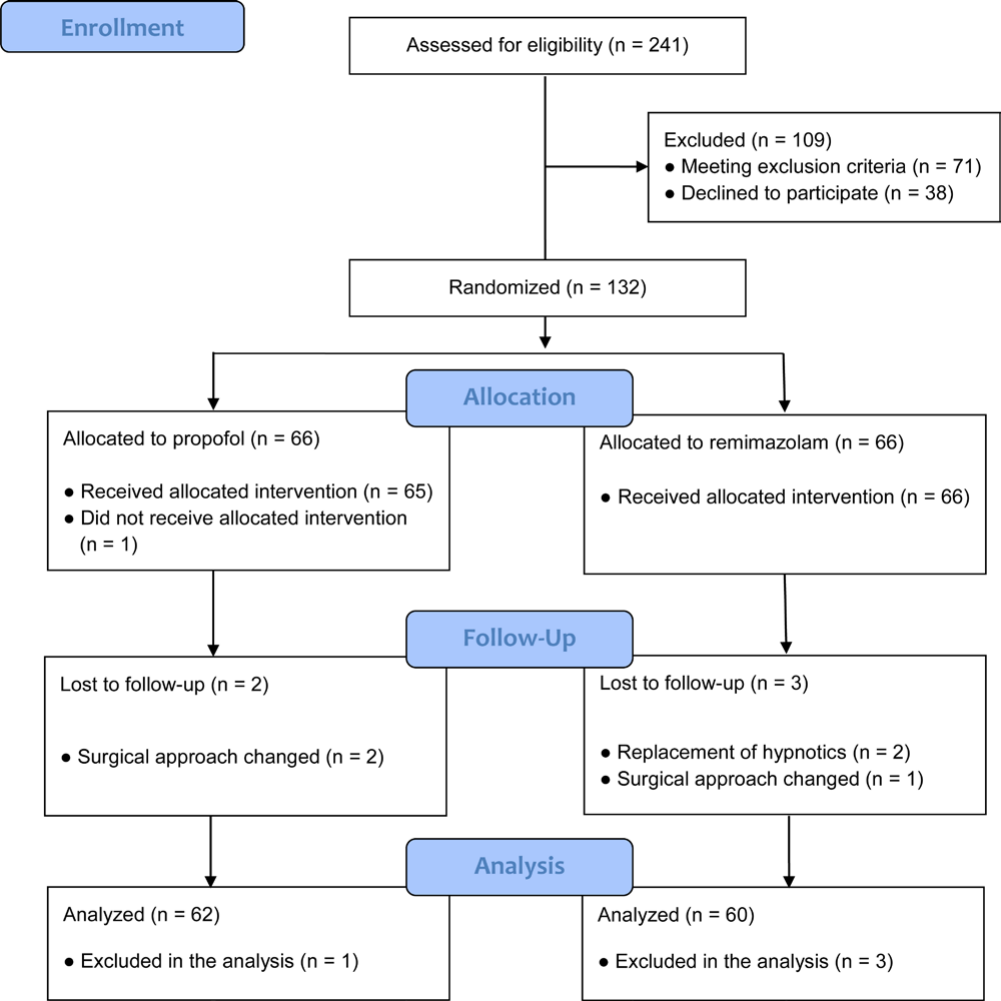
CONSORT flow diagram.

A total of 122 patients were included in the final analysis and 10 patients were withdrawn (Fig. 1). One patient in the propofol group was excluded because of TCI machine malfunction. A patient in the remimazolam group was excluded due to the replacement of the hypnotic agent with sevoflurane at the discretion of the attending anesthesiologist during the initial hypotensive event. Another patient was withdrawn due to BIS > 70 for 10 minutes with tachycardia and hypertension despite the infusion rate of remimazolam > 2 mg/kg/hours and remifentanil effect-site concentration > 5.5 ng/mL, whose sympathetic tone and BIS value of were decreased to the level of stable general anesthesia following the replacement of remimazolam with sevoflurane. Two patients in the propofol group and one in the remimazolam group were excluded because of changes in the surgical method. Case records were lost for 1 patient in the propofol group and 3 patients in the remimazolam group.

The baseline characteristics were comparable in both groups, except for body weight (Table 1). Less than half of the total participants (45.5%) were classified as ASA III. Remifentanil dosage was comparable in the propofol and remimazolam groups (371.2 ± 155.6 vs 404.4 ± 207.5 µg, respectively). Total administered dosage in the groups were 503.0 ± 227.2 mg in the propofol group and 90.8 ± 45.9 mg in the remimazolam group. Flumazenil was administered to 39 patients (65%) in the remimazolam group; 32 patients (53.3%) in the operating room, 4 (6.7%) in the postanesthetic care unit, and 3 (5%) in both the operating room and the postanesthetic care unit. The median dose of flumazenil was 0.2 mg (IQR, 0.2 − 0.4).

**Table 1:** Baseline demographics of the participants.

Variables	Propofol	Remimazolam
	(N = 62)	(N = 60)
Age (yr)	71.5 ± 4.3	70.9 ± 4.3
Female	22 (35.5)	22 (36.7)
Height (cm)	159.9 ± 8.3	160.0 ± 9.4
Weight (kg)*	66.7 ± 9.5	62.9 ± 10.1
Anesthetic duration (min)	77.6 ± 28.8	79.7 ± 27.7
Surgical duration (min)	38.1 ± 28.6	40.3 ± 26.7
Surgery		
Laparoscopic cholecystectomy	39 (62.9)	41 (68.3)
TURBT	23 (37.1)	19 (31.7)
ASA physical status classification		
ASA I	1 (1.6)	1 (1.7)
ASA II	29 (46.8)	36 (60.0)
ASA III	32 (51.6)	23 (38.3)
Baseline HR (beats/min)	73.4 ± 13.8	74.7 ± 15.0
Baseline SBP (mm Hg)	154.2 ± 20.9	155.1 ± 18.9
Baseline DBP (mm Hg)	81.8 ± 10.4	80.6 ± 9.7

Values are presented as mean ± SD or number (%).

* indicates *P* < .05.

DBP = diastolic blood pressure, HR = heart rate, SBP = systolic blood pressure, TURBT = transurethral resection of bladder tumor.

**Figure 2.**
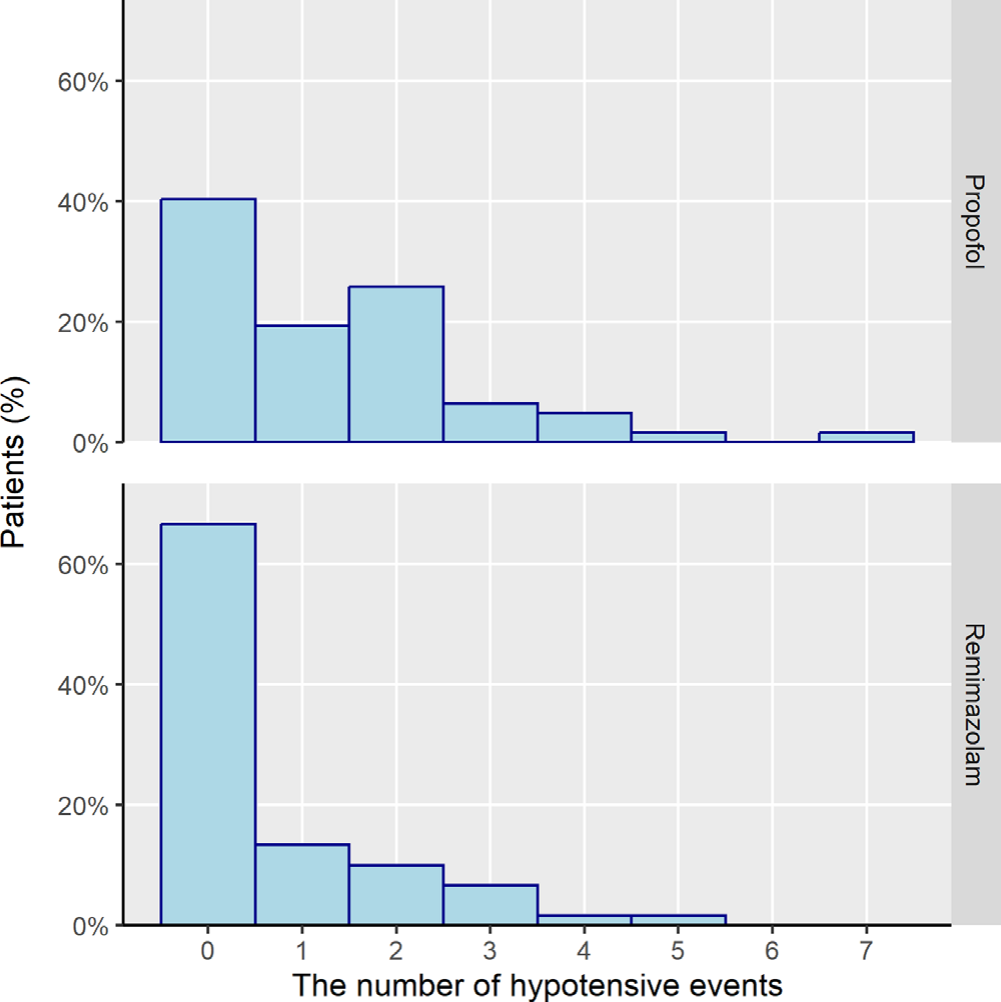
The number of hypotensive events in each patient.

**Figure 3.**
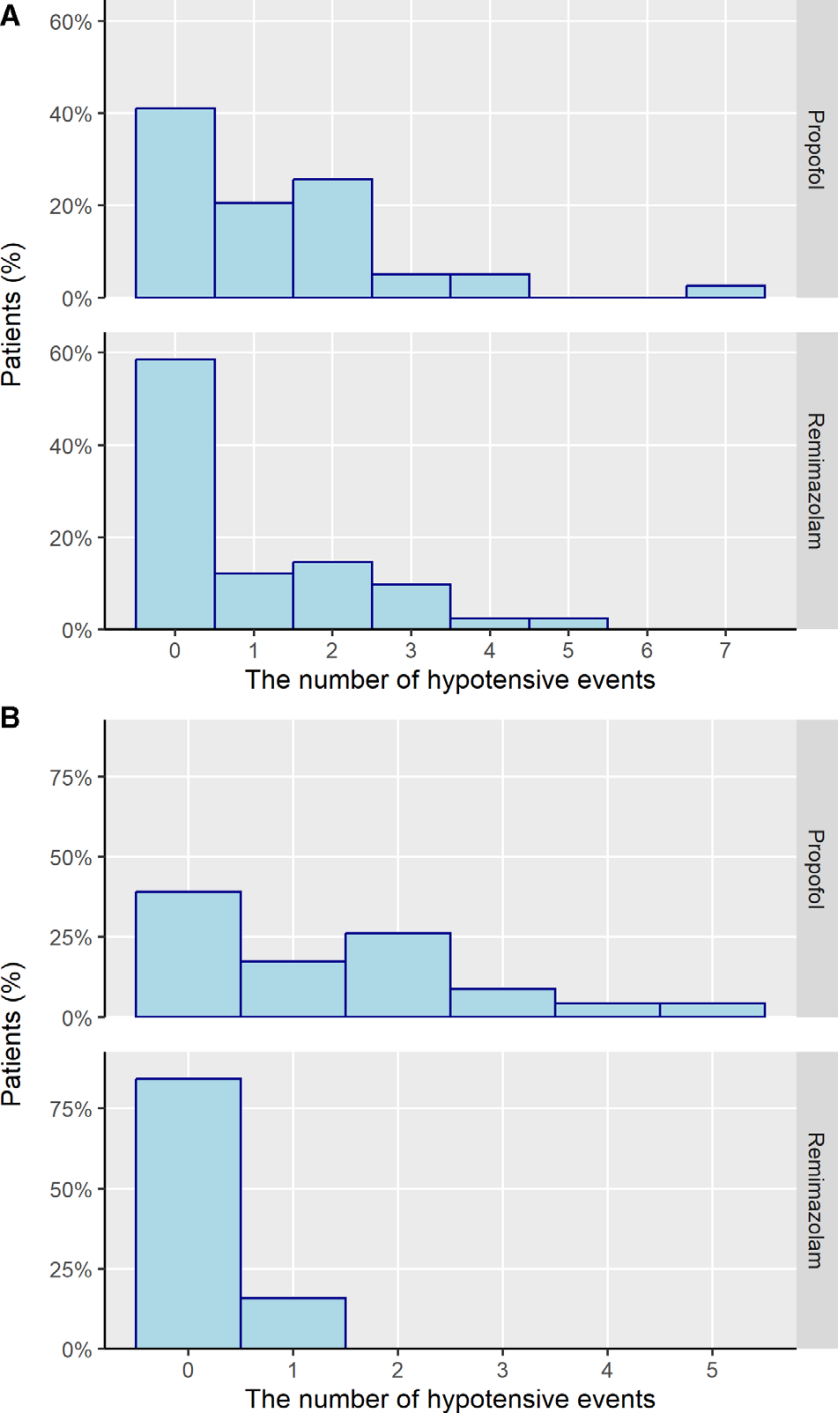
The number of hypotensive events in each patient of subgroup. (A) Laparoscopic cholecystectomy, (B) transurethral resection of bladder tumor.

Fewer patients in the remimazolam group had hypotension compared to those in the propofol group (Table 2, χ^2^ = 7.476, *P* = .006). Hypotensive events were more frequent in the propofol group (W = 2363, *P* = .005; Fig. 2). Subgroup analysis showed the same trends among patients who underwent TURBT, with 45.1% difference in the presence of intraoperative hypotension (Fig. 3). The number of laparoscopic cholecystectomy patients who experienced hypotension was comparable in both groups.

**Table 2:** Incidence of intraoperative hypotension.

	Propofol	Remimazolam	*P* value
	(N = 62)	(N = 60)	
Overall			
Presence of intraoperative hypotension [n, (%)]*	37 (59.7)	20 (33.3)	.006
Intraoperative hypotension events, median (IQR)*	1 (0–2)	0 (0–1)	.005
Laparoscopic cholecystectomy			
Presence of intraoperative hypotension [n, (%)]	23 (59.0)	17 (41.5)	.180
Intraoperative hypotension events, median (IQR)	1 (0–2)	0 (0–2)	.221
TURBT			
Presence of intraoperative hypotension [n, (%)]*	14 (60.9)	3 (15.8)	.008
Intraoperative hypotensive events, median (IQR)*	1 (0–1)	0 (0–0)	.001

* indicates *P* < .01.

IQR = interquartile range, TURBT = transurethral resection of bladder tumor.

Logistic regression analysis revealed a significantly lower odds ratio for experiencing hypotension with remimazolam than with propofol (Table 3). After the adjustment of ASA physical status classification and surgery type, patients in the remimazolam group had a 1 to 3rd lower risk of experiencing intraoperative hypotension.

**Table 3:** Result of logistic regression analysis for intraoperative hypotension.

Variable	Univariate analysis		Multivariate analysis	
	Unadjusted OR (95% CI)	*P* value	Adjusted OR (95% CI)	*P* value
Remimazolam	0.34 (0.16–0.71)	.004	0.34 (0.16–0.73)	.006
ASA physical status classification III	2.04 (0.99–4.20)	.054	2.03 (0.94–4.38)	.072
Surgery (laparoscopic cholecystectomy vs TURBT)	0.68 (0.32–1.45)	.317	0.54 (0.24–1.23)	.145

OR = odds ratio, TURBT = transurethral resection of bladder tumor.

There were no significant differences in secondary outcomes between the groups (Table 4). Postoperative nausea and vomiting were rare in both groups. One patient in the propofol group who underwent laparoscopic cholecystectomy developed sepsis due to an intra-abdominal abscess and required critical care from postoperative day 3. The remaining patients experienced no adverse events.

**Table 4:** Secondary outcomes.

Variables	Propofol	Remimazolam
	(N = 62)	(N = 60)
Administration of rescue antiemetics [n, (%)]	1 (1.6)	1 (1.7)
Time to discharge to the PACU (s)	841.9 ± 213.0	813.8 ± 275.6
Time to extubation (s)	720.8 ± 204.9	671.2 ± 263.1
Intraoperative tachycardia [n, (%)]	14 (22.6)	16 (26.7)
Intraoperative bradycardia [n, (%)]	29 (46.8)	21 (35)
Postoperative delirium (n)	0	0
Postoperative intensive care (n)	1	0
Postoperative mortality in 30 days (n)	0	0
PACU stay [min; (IQR)]	30 (30–30)	30 (30–30)

No statistically significant differences were observed between the groups.

IQR = interquartile range, PACU = postanesthetic care unit.

## 4. Discussion

Intraoperative hypotensive events are risk factors for postoperative mortality in older patients.^[16]^ The results of this study showed a reduced incidence of intraoperative hypotension in older patients following the administration of remimazolam instead of propofol. Other advantages of remimazolam over propofol include reduced airway obstruction, no injection pain, and a drug formulation that is unlikely to support growth of bacteria.^[17]^ Moreover, the risk of intraoperative awareness can be reduced with the administration of benzodiazepines.^[18]^

The recovery profile was similar in patients in both groups. The context sensitive half time of remimazolam is longer than that of propofol.^[17,19]^ However, this difference was nullified by the administration of flumazenil, at least in surgical cases lasting 1 to 2 hours. The recovery time can be even shorter when flumazenil is routinely administered as a reversal agent. In a previous study, the time to extubation after cessation of remimazolam decreased to a value comparable to that after sevoflurane cessation.^[20]^

There was a previous study by Sekiguchi et al^[21]^ comparing remimazolam and propofol during the induction of anesthesia in middle-aged and elderly patients, and no significant difference of hemodynamics were observed between those who administered remimazolam and propofol. There are several difference between the study by Sekiguchi et al and this study. First, the age of target population was different; it ranged from 45 to 80 years old in the prior study and from 65 to 80 years old in this study. Second, while the previous study compared hypotension during the induction of anesthesia, the current study examined intraoperative hypotension from the induction to the conclusion of anesthesia. Third, the doses of induction agents differed The propofol effect-site concentrations were 3 μg/mL in the previous study and 4 μg/mL in the current study, and the loading dose of remimazolam was 12 mg*kg^-1^*hours^-1^ in the prior study and 6 mg*kg^-1^*hours^-1^ in the current study. It is worth noting that in the study by Sekiguchi et al, patients in the remimazolam group lost consciousness within 3 minutes, while about half of those administered propofol took 4 minutes or more to lose consciousness.

Remimazolam could be adopted as a reasonable alternative hypnotic agent to propofol, especially in patients vulnerable to decreased blood pressure. Some anesthesiologists may be reluctant to use remimazolam because of the risk of perioperative delirium associated with benzodiazepines. However, none of our patients administered remimazolam developed delirium. Similarly, the drug was not reported to be associated with delirium in patients undergoing cardiovascular surgery or transcatheter aortic valve implantation.^[22,23]^

A lower dose of remimazolam is required for LOC in older patients and adjusting the remimazolam dose can further lower the risk of hypotension.^[24–26]^ Dextran 40, a stabilizer in the remimazolam preparation, is associated with non-IgE-mediated hypersensitivity. Reducing the infusion rate can help in controlling hypotension following administration of remimazolam.^[27]^

The study limitations include the type of surgery and possibly the single ethnicity of the participants. However, the race is expected to have a small to negligible effect on pharmacological profile of remimazolam.^[28]^ Furthermore, randomized controlled trials which included other surgeries showed less hemodynamic perturbation in patients administered remimazolam as compared to those administered propofol.^[29,30]^ Further studies involving patients with other comorbidities are required to elucidate the safety profile of remimazolam.

## Author contribution

**Conceptualization:** Yeong-Gwan Jeon, Ji-Hyoung Park, Seung Woo Song.

**Data curation:** Jonghoon Lee, Sang A Song.

**Funding acquisition:** Yeong-Gwan Jeon.

**Formal analysis:** Ji-Hyoung Park, Seung Woo Song.

**Investigation:** Yeong-Gwan Jeon, Sujin Kim, Seung Woo Song.

**Methodology:** Yeong-Gwan Jeon, Seung Woo Song.

**Project administration:** Yeong-Gwan Jeon, Seung Woo Song.

**Supervision:** Yeong-Gwan Jeon, Hyun Kyo Lim, Seung Woo Song.

**Validation:** Ji-Hyoung Park, Seung Woo Song.

**Visualization:** Seung Woo Song.

**Writing – original draft:** Yeong-Gwan Jeon, Sujin Kim, Seung Woo Song.

**Writing – review & editing:** Yeong-Gwan Jeon, Ji-Hyoung Park, Jonghoon Lee, Sang A Song, Hyun Kyo Lim, Seung Woo Song.
